# The impact of disability-related deprivation on employment opportunity at the neighborhood level: does family socioeconomic status matter?

**DOI:** 10.3389/fpubh.2023.1232829

**Published:** 2023-08-09

**Authors:** Ning Qiu, Yuxiao Jiang, Zongyao Sun, Mengbing Du

**Affiliations:** ^1^School of Architecture and Urban Planning, Shandong Jianzhu University, Jinan, China; ^2^Department of Architecture and Civil Engineering, City University of Hong Kong, Kowloon, Hong Kong SAR, China; ^3^School of Architecture, Tianjin University, Tianjin, China; ^4^School of Political Science & Public Administration, Wuhan University, Wuhan, China; ^5^Local Government Public Service Innovation Research Center, Wuhan University, Wuhan, China

**Keywords:** disability-related multiple deprivations, employment opportunity, family socioeconomic status, disabled population groups, well-being

## Abstract

**Introduction:**

Difficulties in attaining employment significantly contribute to socioeconomic poverty among individuals with disabilities. However, our understanding of how socioeconomic deprivation experienced by individuals and families with disabilities influences employment opportunities remains incomplete. This study aims to explore the relationship between index of disability-related multiple deprivation (IDMD) and employment opportunities (EMPO), while also investigating the role of family socioeconomic status (FSES) in shaping this relation.

**Methods:**

This study explores the heterogeneous effects of IDMD, FSES, and the interaction between IDMD*FSES on EMPO among four disabled population groups categorized by IDMD and FSES.

**Results:**

Results reveal that IDMD has a significant negative impact on EMPO, suggesting that persons with disabilities are confronted with a poverty trap resulting from the relationship between IDMD and EMPO. Furthermore, FSES demonstrates an effective moderating role in the IDMD-EMPO relationship, with the greatest impact observed among disabled population groups characterized by high IDMD and low FSES.

**Discussion:**

The findings suggest that family-level support is crucial for vulnerable groups of disabled individuals to overcome the poverty trap, surpassing the reliance on individual-level assistance alone. This study supports a paradigm shift in comprehending disability-related deprivation by acknowledging its association with families, thereby presenting opportunities to enhance the welfare of people with disabilities.

## 1. Introduction

Employment can enhance social sustainability and individual well-being (1–3). Specifically, for individuals with disabilities, gaining employment represents a significant opportunity that not only promotes their economic sustainability but also fosters their social participation (4). According to global statistics, individuals with disabilities have an employment rate of 44%, which is significantly lower than 75% among those without disabilities (3). This significantly low employment rate among disabled individuals adversely affects both their welfare and society as a whole (5). Employment prospects for individuals with disabilities are typically confined to sectors like retail, customer service, manufacturing, and assembly, which often offer low wages and job security (6). Although there are cases where individuals are referred to non-profit organizations and social service agencies for employment assistance under social welfare policies, such opportunities are frequently limited in both scope and availability. Unemployment among persons with disabilities contributes to various social issues, including limited labor market participation, increased poverty rates, and diminished self-esteem (7–9).

Previous studies have explored the impact of physical, social, and environmental factors on the employment prospects of disabled individuals (4). The employment opportunities for persons with disabilities are often constrained by their physical condition, such as the severity of their disability and overall health status. Generally, individuals with more severe disabilities encounter more significant challenges in attaining and retaining employment than those with milder disabilities (10). Additionally, persons with disabilities often face negative stereotypes and labels, deemed unqualified, unproductive, or costly to hire (11). Environmental challenges encountered by disabled individuals include a lack of barrier-free services (12). However, existing studies have primarily focused on either the internal or external factors that affect the employment of disabled individuals. In comparison, there is a scarcity of research examining the relationship between deprivation, poverty, and the employment prospects of people with disabilities from a multidimensional perspective (12–14). Recognizing and understanding this relationship is crucial, as it offers policymakers valuable insights for enhancing the well-being of individuals with disabilities by exploring the complex interplay between deprivation, poverty, and employment opportunities, enabling the development of targeted interventions and policies to address their unique challenges and improve their overall quality of life.

Additionally, family plays a crucial role in studies related to poverty, as family socioeconomic status is closely linked to the perpetuation of poverty and the exacerbation of social polarization (15, 16). Within a family context, parents’ educational attainment and socioeconomic status are strongly associated with their offspring’s job opportunities and income distribution (17). For individuals with disabilities, family support assumes even greater significance, as they often require additional healthcare, financial guidance, and counseling from their families (9, 18, 19). In many cases, immediate family members, including parents, siblings, or spouses, often serve as primary caregivers for individuals with disabilities, residing in the same household and providing crucial daily care, mobility support, emotional assistance, medication management, and advocacy. Their role significantly enhances the well-being and quality of life of individuals with disabilities. This implies that a supportive family environment can mitigate the physical and social disadvantages faced by disabled individuals at the individual level. However, limited attention has been given to exploring the impact of family socioeconomic status on disability employment and the potential moderating effects of such factors on the relationship between deprivation and employment. Investigating the impact of family status is crucial both theoretically and practically. Theoretically, it extends and reinforces the theories of intergenerational poverty and the vicious cycle of poverty. On a practical level, it presents an opportunity to identify interventions that can enhance the well-being of people with disabilities.

This study aims to address these research gaps by examining the relationship between deprivation and employment, while also investigating the moderating influence of family socioeconomic status on this relationship. The remainder of this study is structured as follows. Section 2 introduces the theoretical framework and presents the research hypothesis. Section 3 outlines the research methodology, including the study area, measurement instruments, data processing techniques, and statistical models employed. Section 4 presents the empirical findings of the study. Section 5 offers a comprehensive discussion of the implications of the deprivation-employment relationship. Finally, Section 6 concludes the study by summarizing the key findings, providing policy recommendations, and suggesting avenues for further research and development.

## 2. Theoretical basis and hypothesis

### 2.1. Disability-related deprivation and disability employment gap

The disability employment gap, which refers to the disparity in employment rates between disabled and non-disabled individuals, serves as a crucial measure of disability equality (20). Persons with disabilities often face additional forms of deprivation, such as social stigma, challenges in rehabilitation, or difficulties in accessing assistive technology (21). Extensive research has been conducted to understand the underlying mechanisms that contribute to the disability employment gap and to drive policy changes. These studies typically consider both internal and external factors to elucidate these mechanisms.

Internal factors, such as health-related deprivation, particularly body or functional impairments, can significantly impact individuals’ social engagement, participation, mobility, and competency in the job market (22). In many workplaces, deviations from the norm, including illness-related participation limitations, are often met with limited acceptance (23). Furthermore, workers with disabilities tend to possess lower skill levels due to fewer educational and training opportunities. Individuals with special needs typically acquire fewer qualifications than those without (24).

In addition to internal factors such as job readiness, medical benefits, and academic attainment, scholars have identified various external indicators that contribute to the employment gap among people with disabilities. These external factors include employer attitudes, transportation accessibility, and neighborhood characteristics (25, 26). Firstly, social stigma continues to impact the work experiences of individuals with disabilities significantly. They face unequal employment opportunities, higher levels of job discrimination, and lower levels of support from supervisors and coworkers (10). Although disability does not inherently limit job productivity, applicants with disabilities often experience discrimination from employers (27). Secondly, physical barriers, long commutes, and a lack of barrier-free facilities create significant challenges for individuals with disabilities in entering the workforce (28). While providing accommodations can help overcome these challenges, seeking accommodation may also reduce employment opportunities (29, 30). Considering the above discussions, we propose *H1* as follows:

*Hypothesis 1 (H1)*: Disability-related deprivation negatively impacts employment opportunities for individuals with disabilities.

### 2.2. Moderating role of family socioeconomic status on deprivation-employment relations

In recent decades, there has been a significant shift from a deficit-oriented approach to a supportive paradigm in understanding disability. While individual-level support has received considerable attention, the importance of family-level support has also been recognized in policy considerations (31). Consequently, a growing body of literature has focused on the role of family support for individuals with disabilities (32–35). Family-level support encompasses various aspects, including emotional support, socioeconomic status, caregiving, quality of life, and marital quality (32, 34, 36). Among these forms of support, family socioeconomic status stands out as a crucial determinant in shaping the levels of disability-related deprivation across three primary domains.

Firstly, socioeconomic status plays a significant role in predicting various health outcomes (37, 38). Individuals from impoverished backgrounds are more prone to experiencing disabilities due to factors such as malnutrition, which is strongly associated with stunting and the onset of disabilities (39). Additionally, economically disadvantaged families often face restricted living and working conditions (40), resulting in unemployment and loss of income (41).

Secondly, family characteristics significantly influence the social benefits experienced by individuals with disabilities through various pathways. First and foremost, it is undeniable that the socioeconomic status of families with individuals with disabilities directly influences the level of financial support provided to persons with disabilities (42). Additionally, the socioeconomic status of the family is the strongest predictor of an individual’s educational performance (43, 44). Moreover, family socioeconomic status can shape the characteristics of an individual’s social network, which, in turn, provides opportunities for social participation and access to resources (37, 45).

Thirdly, the socioeconomic status of a family determines the level of accessibility to various resources in their residential area (46, 47). An accessible environment is one of the essential elements of rehabilitation. The availability of assistive services and a barrier-free environment may greatly improve the mobility and independence of persons with disabilities (48). This, in turn, supports the provision of equitable employment opportunities for disabled individuals (49). Accordingly, we propose *H2*, as shown below.

*Hypothesis 2 (H2)*: Family socioeconomic status positively moderates the deprivation-employment relations.

### 2.3. Heterogenous characteristics amongst the disabled population groups

Recognition of the diverse subgroups within the disabled population has gained increasing attention, leading to a more nuanced understanding of disability (50). Research has focused on disaggregating disability groups to address specific disability issues and develop targeted disability policies that account for their heterogeneity. Many studies have explored the demographic characteristics of disabled individuals, such as gender, age, severity levels of disability, and types of disabilities, to identify subgroups that are more vulnerable to poverty and deprivation (51–53). While these studies emphasize the prevention of disease and deprivation, there is limited knowledge about early preventative strategies to mitigate poverty and deprivation among disabled individuals.

On the one hand, the socioeconomic status of families with individuals with disabilities is often affected by the lower income levels and higher expenses associated with caring for disabled family members (54). On the other hand, considering the family-dependency characteristics of disabled individuals, the influence of family-level characteristics on their outcomes extends throughout their lifetime (36, 55). Therefore, similar to individual-based differences, the heterogeneity within the disabled population should also be considered at the family level, as family factors play a significant role in shaping individual development (56). In this context, it is essential to consider individual factors (such as gender and age) related to the individual’s health condition and family factors (such as economic conditions and household assets) in understanding the social context surrounding disabled individuals. Examining the heterogeneity of individual factors provides insights into addressing diverse disability issues while analyzing the heterogeneity of family factors allows for the formulation of targeted policy strategies to enhance the well-being of disabled individuals. Based on the discussions, we develop *H3* as shown below.

*Hypothesis 3 (H3)*: The impact of family socioeconomic status is heterogenous amongst different disabled population groups.

## 3. Research strategies

### 3.1. Study area

**Figure 1:**
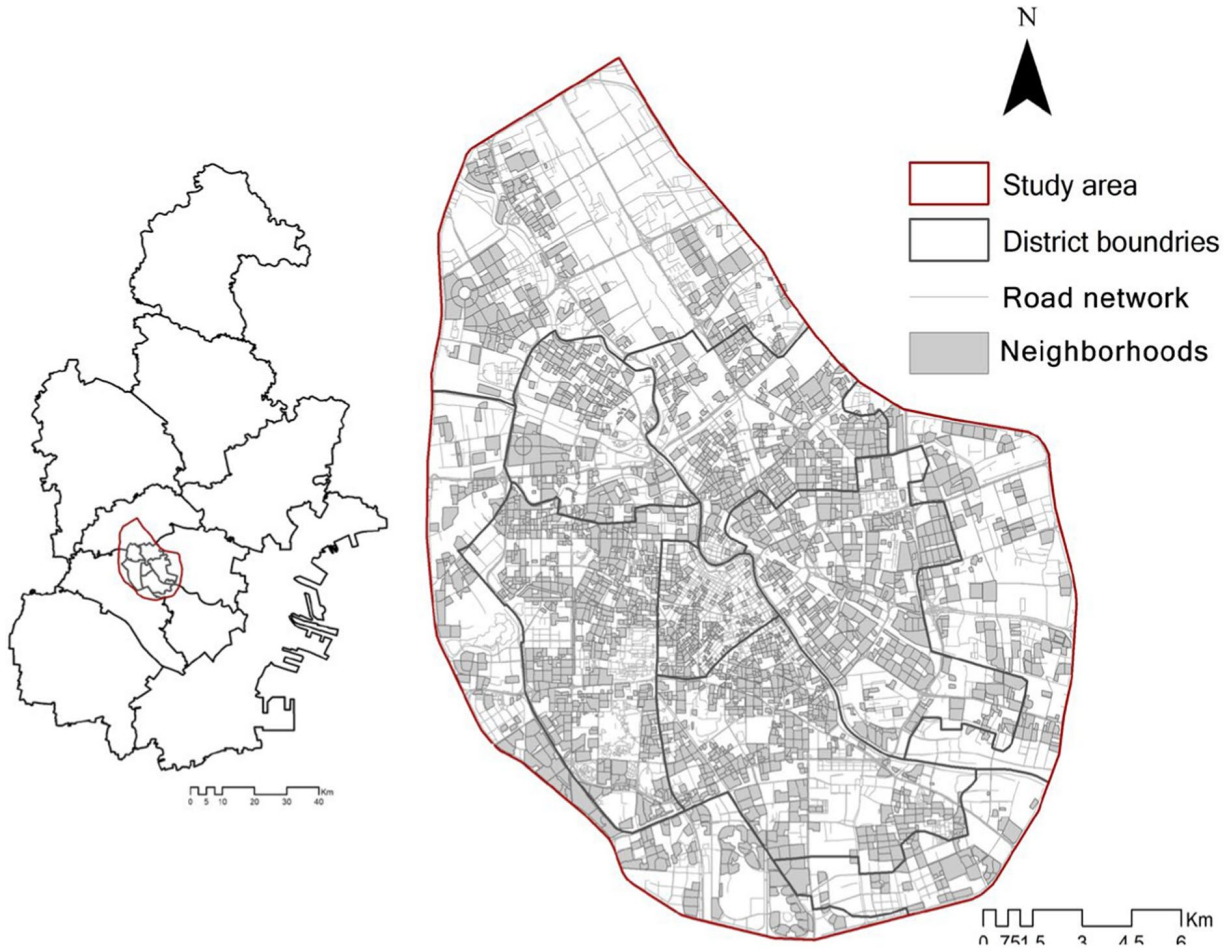
Study area.

The study area selected for this research is Tianjin, an important industrial megacity located southeast of Beijing in China. Figure 1 depicts the geographical extent of the study area, which spans a land area of 576 km^2^, accounting for 13.3% of the total land area in Tianjin. The population of disabled individuals in the study area is 177,000, representing 50.6% of the total disabled population in Tianjin, as recorded in the Tianjin Disability Database.

The study area consists of 2,484 neighborhoods, including commodity neighborhoods, danwei housing, and subsidized neighborhoods. Most of these housing units are gated communities characterized by intense social interaction, strong social cohesiveness, and relatively less pronounced social inequality (57). The average neighborhood size in the study area is 0.042 km^2^, with an average of 11 buildings and 855 houses within each neighborhood. The number of disabled individuals in each neighborhood ranges from 0 to 2,005, with an average of 54 disabled individuals per neighborhood.

### 3.2. Measurements

#### 3.2.1. Measures of employment opportunity (EMPO)

Employment opportunities are commonly measured using employment accessibility, as it is widely recognized as a measure of “the potential for opportunities for interaction” (58, 59). This concept implies that employment opportunities will likely increase with improved accessibility and favorable location opportunities (60).

In China, there are four primary forms of employment for individuals with disabilities: (1) Proportional employment (PE), where employers allocate job positions for disabled individuals in a specific proportion; (2) Intensive employment (IE), which involves placement in welfare enterprises, therapeutic and industrial establishments, sheltered factories, and similar settings; (3) Flexible employment (FE), allowing for self-employment or entrepreneurship; (4) Assisted employment (AE), is a sheltered, non-profit, social welfare-oriented form of employment designed for individuals with intellectual, mental, and severe physical disabilities. Disability employment facilities play a significant role in all four employment types. Firstly, these facilities provide PE and AE workplaces that offer disabled individuals the necessary equipment, tools, and welfare amenities. They create a conducive physical environment that enables disabled persons to work in a supportive and inclusive setting. Secondly, these facilities not only serve as a means to access PE and FE opportunities but also provide access to employment-related resources and support for individuals with disabilities. This includes services such as employment training and counseling to enhance their skills and facilitate their successful integration into the workforce. To characterize employment opportunities in this study, the accessibility to disability employment facilities is calculated using the following formula:


(1) $$A_{i}=\sum_{j=1}^{n}\frac{M_{j}}{D_{ij}{\;}^{\beta }V_{j}}\;$$

$$V_{j}=\sum_{k=1}^{m}\frac{P_{i}}{D_{ij}^{\beta }}$$

$A_{i}$ is the disability employment facility accessibility in neighborhood *i*, $M_{j}$ the total number of job opportunities at a facility, which is derived from the capacity attribute of the facility data set. $D_{ij}$ is the cost of time for disabled people to access facilities. $V_{j}$ is the population size factor, which considers the spatial distribution of the disabled population in the vicinity of a facility, reflecting competition for limited resources resulting from the use of the same facility by the users. *m* is the number of neighborhoods, $P_{i}\;$ is the disabled people in the neighborhood *i*, *β* is the travel friction, which was set to 1.5 for a power function.

#### 3.2.2. Measures of index of disability-related multiple deprivation (IDMD)

In a study by Qiu et al., an Index of Disability-related Multiple Deprivation (IDMD) was developed at the sub-district level, utilizing six specific domains to assess disability-related deprivation (12). The IDMD incorporates the domains of income, marital status, education, health, and services to define multiple deprivation (Table 1). The weights assigned to each domain are as follows: 25% for income, 15% for marital status, 25% for education, 15% for health, and 20% for services, based on measurements of deprivation (61, 62). Within each domain, the indicators are given equal weight (Table 1). A higher IDMD score indicates a higher level of multiple deprivation. The IDMD index is calculated based on the formula as shown below:


(2) $$\begin{array}{l} \text{IDMD}=0.2*\text{emplyment}+0.15*\text{income}+0.15*\text{marital status}\\ \;+0.2*\text{healht}+0.2*\text{services} \end{array}$$

**Table 1:** The domains and indicators of the IDMD.

Domain	Domain weight (%)	Indicators	Indicator weight (%)
Income	25	% industrial worker in total employees with disabilities	12.5
		% lower social-services workers in total employees with disabilities	12.5
Marital status	15	% disabled people (aged >20) who have never been married	7.5
		% divorced disabled people	7.5
Education	25	% disabled people without a diploma	12.5
		% disabled people (over15 years old) with lower educational attainment (under junior high school)	12.5
Health	15	% people with the most severe level^a^ of disability	15
Services	20	∑closest distances of each type of disability services facilities to sub-district population weighted centroid	10
		∑closest distances of each type of basic public services facilities to sub-district population weighted centroid	10

a Each type of disability is divided into four levels depending on their severity, according to the “*Practical Assessment Standards for People with Disabilities in China*.” The most severe level (1^st^ level), whatever the disability type is, means very serious barriers to participation in social life.

#### 3.2.3. Measures of family socioeconomic status (FSES)

FSES is often measured by parents’ education level, parents’ occupation, family income (63–65), or family resources (e.g., family possessions) (66, 67). In China, real estate is crucial as it provides a physical space to fulfill people’s basic survival needs and serves as a direct material asset for families. The China Household Wealth Survey Report (2018) indicates that real estate accounts for 71.35% of household wealth, making it a significant indicator of a family’s wealth and social status (68).

This study collects real estate data, which encompasses information on housing and neighborhood characteristics. The collected data includes the following variables: (1) housing price, (2) housing age, (3) management costs, (4) greenery cover, and (5) service facilities, such as healthcare, education, transportation, parks, and shopping. Table 2 provides detailed information on the variables related to real estate data.

**Table 2:** Indicators of housing and neighborhood characteristics measuring FSES.

Domain	Indicators	Definition of indicators	Mean	Std. Dev.	Min	Max
Housing	Housing value	Average housing price per square meter (RMB/m^2^)	28,946	13,934	10,277	102,161
	Housing age	The construction time of building (years)	24.36	7.76	2	39
	Management costs	Average management fee per square meter (RMB/m^2^)	1.07	1.02	0.1	8
	Greenery cover	Percentage of total green space (%)	25.6	8.67	0.13	0.65
Neighborhood	Healthcare	Number of health services within 3 km	36	19.66	0	78
	Education	Number of education services within 3 km	88	50.22	0	139
	Transport	Number of public transport stations within 3 km	71	24.28	0	136
	Park	Number of parks within 3 km	22	14.49	0	60
	Shopping	Number of commercial services within 3 km	860	371.89	0	1,585

This study used principal component analysis (PCA) to determine the weight for each indicator to measure housing characteristics, attempting to reduce error and subjectivity. Housing characteristics measuring FSES is calculated using Eq. 3 (69):


(3) $$\text{FSE}S_{i}=\sum_{i=1}^{n}E_{i}\times \left(\sum_{j=1}^{k}L_{j=1}\times x_{j}\right)$$

Where $E_{i}$ is the eigenvalue of component I; $L_{i}\;$ is the loading score for indicator j; $x_{j}$ is the standardized value of indicator *j*. All indicators were in the same direction. In each spatial unit (neighborhood), the weight of each domain was multiplied by each indicator, and the FSES value was calculated by adding all the products together. A choropleth map was created to visualize the geographical distribution of FSES by quintile.

#### 3.2.4. IDMD-FSES disabled population groups

**Figure 2:**
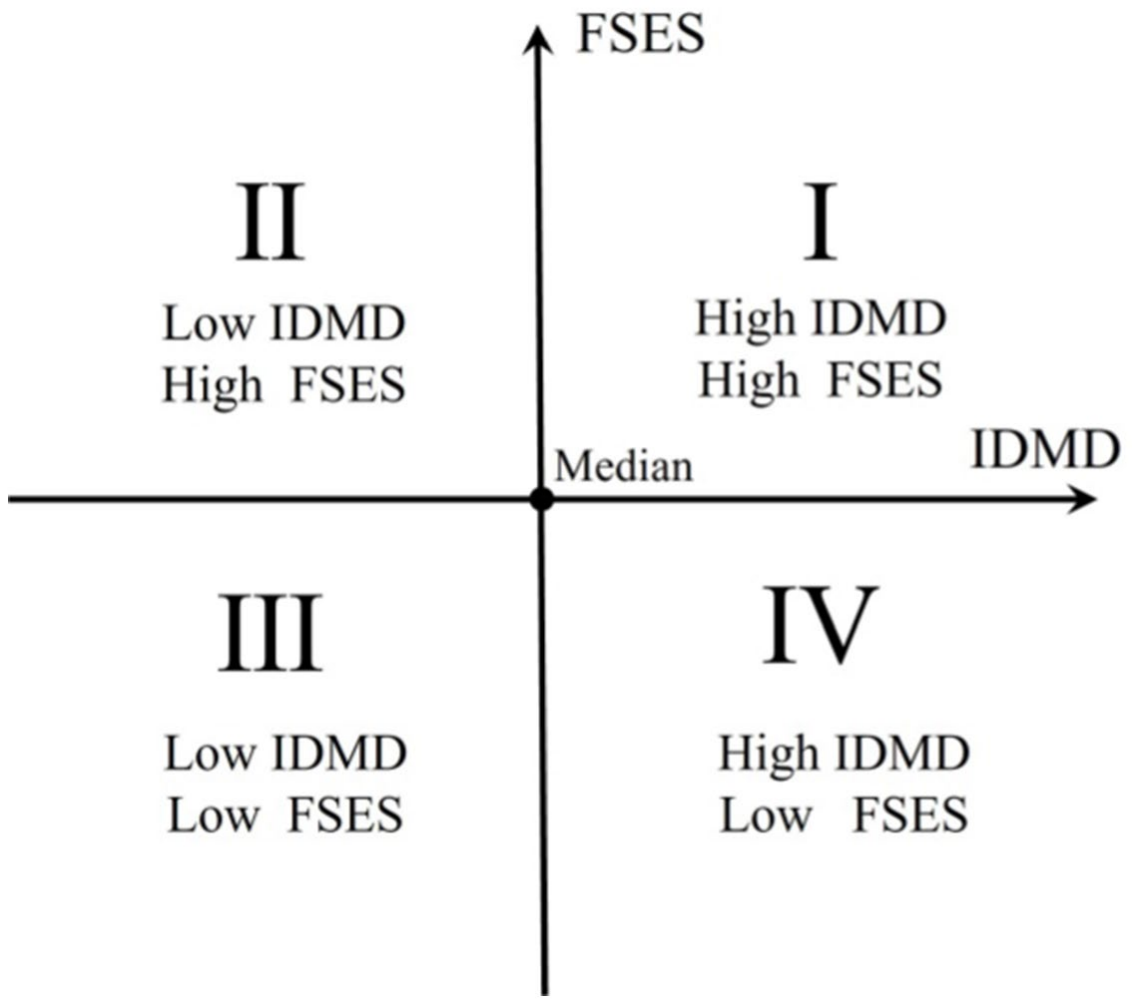
Four quadrants diagram of IDMD-FSES disabled population groups.

The four-quadrant clustering (FQC) method (70) categorizes the disabled population into four distinct groups based on their individual and family conditions. These groups are defined as follows: First Quadrant: This group comprises individuals with poor individual conditions but better family conditions (high IDMD − high FSES); Second Quadrant: This group consists of individuals with both better individual and family conditions (low IDMD − high FSES); Third Quadrant: This group includes individuals with better individual conditions but low-income family conditions (low IDMD − low FSES); Fourth Quadrant: This group is characterized by individuals with both low-income individual and family conditions (high IDMD – low FSES) (Figure 2).

### 3.3. Data and processing

This study uses three primary data sources. Firstly, the social and health attributes of certified disabled people are collated from the Tianjin Disability Database 2020. It contains personal data (gender, age, disability type, disability level, household registration, present address, workplace, education, and marital status) of those disabled people across Tianjin who meet the national criteria for issuing disability cards. Secondly, the location and descriptors (type, year of construction, area, medical and nursing personnel numbers) of 500 disability service facilities (60 disability schools, 300 disabilities daycare centers, 94 rehabilitation centers, 7 integrated disabled services, and 44 disability employment services) are collated from Database of Disabled Facilities 2020 in Tianjin. The two types mentioned above of data are provided by the Tianjin Disabled Persons’ Federation (TDPD).^1^ Thirdly, the data of 2,484 neighborhoods, which represent family socioeconomic status (FSES), are drawn from the second-hand housing information of Fang.com.^2^ This study aggregated data to the neighborhoods level, and the research variables were summarized in Table 3.

**Table 3:** Description of research variables.

Variable	Abbreviation	Obs.	Mean	Std. Dev.	Min	Max
Employment opportunity	EMPO	2,484	0.166	0.094	0	0.457
Index of disabled-related multiple deprivation	IDMD	2,484	0.311	0.056	0	0.744
Family socioeconomic status	FSES	2,484	28,946	13,934	10,277	102,161
% Male disabled people	GEN	2,484	0.520	0.561	0.133	1
% Labor population	PLAR	2,484	0.386	0.169	0	1.142
% Urban Hukou	HK	2,484	0.97	0.153	0	1
Population of neighborhood	POP	2,484	548.55	506.97	10	4,036
% Disabled people	PDIS	2,484	0.381	1.718	0	26.1

### 3.4. Research models

#### 3.4.1. Spatial distribution analysis

This study employs spatial models based on ArcGIS 10.2 for spatial analysis. The boundaries of the 2,484 neighborhoods are obtained through image mapping and further refined using address information. ArcGIS allows for creating choropleth maps, which are used to visualize the geographical distribution of three variables: EMPO, IDMD, and FSES. The values of these variables are classified into quintiles, and the choropleth maps represent the distribution pattern of each variable across the neighborhoods. This visualization technique helps to convey important information about the data and provides a clearer understanding of the spatial patterns and trends in the study area.

#### 3.4.2. Regression analysis models

##### 3.4.2.1. Phase 1: impact of IDMD on EMPO

Phase 1 adopts OLS model to investigate the impact of IDMD on EMP. The model is specified as Eq. 4


(4) $$\begin{array}{l} \text{EMPO}=\alpha_{0}+\alpha_{1}\text{IDMD}+\alpha_{2}\text{FSES}+\alpha_{3}\text{GEN}\\ \;+\alpha_{4}\text{PLAR}+\alpha_{5}\text{HK}+\alpha_{6}\text{POP}+\alpha_{7}\text{PDIS}+\varepsilon \end{array}$$

Where EMPO is employment accessibility; *IDMD* refers to disabled-related deprivation. *FSES* is family socioeconomic status. GEN represents gender; PLAR is the percentage of labor population; HK is Hukou; POP refers to population; PDIS denotes the percentage of disabled people. ε is the residual term. Considering the different units and scales of variables, all variables are involved in the logarithmic form.

##### 3.4.2.2. Phase 2: moderating effect of FSES on IDMD-EMPO relations

Phase 2 employs moderating effect mode to test whether FSES could moderate IDMD-FSES relations, shown as Eq. 4.


(5) $$\text{EMPO}=\gamma_{0}+\gamma_{1}\text{IDMD}+\gamma_{2}\text{FSES}+\gamma_{3}\text{IDMD}*\text{FSES}+\gamma_{4}\sum X+\varepsilon$$

Where X denotes control variables including GEN, PLAR, HK, POP, and PDIS. The interaction terms evinced in Eq. 2 manifest the moderating effects of *FSES*.

##### 3.4.2.3. Phase 3: heterogenous effect analysis of IDMD, FSES, and IDMD*FSES on EMPO

Phase 3 aims to investigate the heterogenous effect of *IDMD*, *FSES*, and IDMD*FSES on *EMPO* of four *IDMD-FSES* disabled population groups, respectively. The formula applied is similar to Eq. 5.

## 4. Empirical results

### 4.1. A spatial description of employment opportunity, disability-related deprivation, and family socioeconomic status in Tianjin

**Figure 3:**
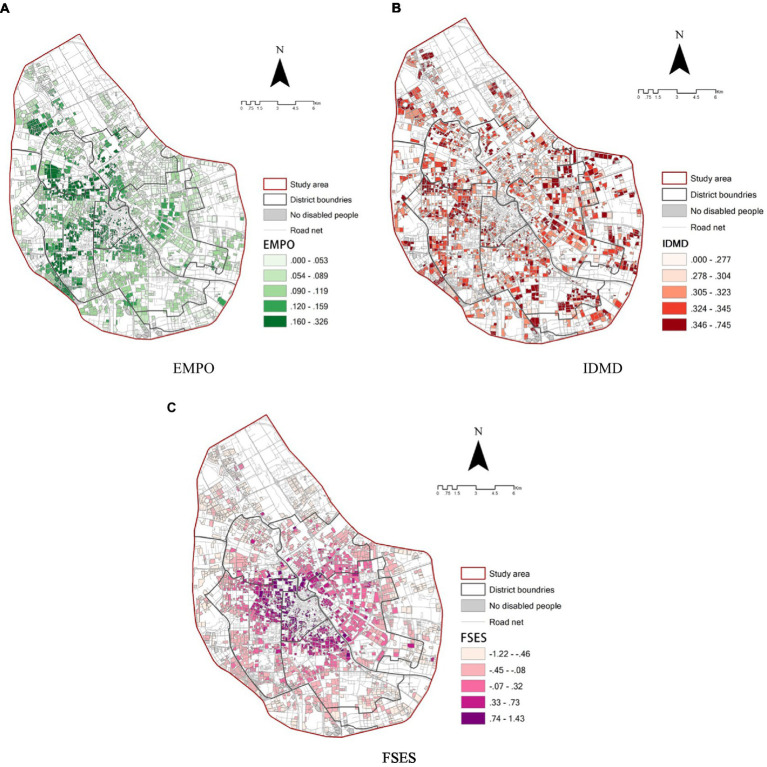
The spatial pattern of **(A)** EMPO, **(B)** IDMD, **(C)** FSES at the neighborhood level.

Figure 3A shows the spatial pattern of EMPO at the neighborhood level in the Central Urban Area of Tianjin. The employment accessibility is categorized into five classes using equal intervals in ArcGIS, with the lowest range being 0–0.053 and the largest range being 0.160–0.326. Overall, EMPO has a pattern characterized by higher values in the west and center of the study area.

The spatial pattern of IDMD is shown in Figure 3B. Similarly, the IDMD is categorized into five classes, with the lowest range of 0–0.277 and the highest range of 0.346–0.745. From the spatial perspective, areas with low levels of IDMD are clustered in the center, whilst areas with a high level of IDMD are located in the west and east.

Different patterns are found in terms of the distribution of FSES (Figure 3C). Overall, there is an uneven distribution of FSES with a strong geographical pattern: the agglomeration of high levels of FSES has gone “from center to perimeter.” This suggests that residents in the center have better family socioeconomic backgrounds than those in the periphery.

Figure 3 overall suggests a correlation in the relationship between EMPO, IDMD, and FSES: the high levels of EMP are in high levels of FSES and low levels of IDMD but are also elevated to some degree in the West.

### 4.2. Main effect analysis: employment opportunity in relation to disability-related deprivation and family socioeconomic status

Table 4 shows the main effects of the explanatory variables (IDMD and FSES) on EMPO. The results of Models (3)–(8) demonstrate through stepwise regressions that the results remain robust with the inclusion of control variables.

**Table 4:** Results of the baseline regression.

	(1)	(2)	(3)	(4)	(5)	(6)	(7)	(8)
IDMD	−1.187***		−1.437**	−1.509**	−1.684**	−1.631**	−1.631**	−1.636**
	(0.316)		(0.657)	(0.659)	(0.659)	(0.657)	(0.657)	(0.656)
FSES		2.430***	1.650***	1.619***	1.543***	1.530***	1.532***	1.536***
		(0.0549)	(0.365)	(0.366)	(0.366)	(0.364)	(0.365)	(0.364)
GEN				−0.0411	0.0164	0.0133	0.0136	0.00451
				(0.0333)	(0.0360)	(0.0359)	(0.0360)	(0.0362)
PLAR					−0.105***	−0.102***	−0.102***	−0.0942***
					(0.0257)	(0.0257)	(0.0258)	(0.0260)
HK						0.241***	0.240***	0.238***
						(0.0613)	(0.0614)	(0.0613)
POP							0.00154	0.0143
							(0.00850)	(0.0100)
PDIS								0.0185**
								(0.00772)
Constant	−1.629***	−3.087***	−2.699***	−2.704***	−2.707***	−2.692***	−2.700***	−2.729***
	(0.0863)	(0.0277)	(0.180)	(0.180)	(0.179)	(0.179)	(0.185)	(0.186)
Observations	2,416	2,416	2,416	2,416	2,416	2,416	2,416	2,416
R-squared	0.006	0.448	0.449	0.449	0.453	0.457	0.457	0.458

The asterisks ***, ** represent 1%, 5%, and 10% of significance levels, respectively. The numbers in parentheses are the corresponding standard errors.

According to the results, IDMD exerts significant and negative effects on EMPO with a coefficient of −1.636 at the value of *p* < 0.05. The negative association indicates that the higher level of IDMD that a disabled person has, the lower his EMPO. Accordingly, *H1* is supported. Specifically, a 1% unit increase in IDMD can lead to a −1.636% decline in EMPO. The findings indicate that mitigating deprivation and improving employment among disabled people are largely compatible.

Another key variable, FSES, significantly influences EMPO with a coefficient of 1.536 (*p* < 0.01). The results imply that rising FSES is correlated with greater EMPO: an increase of 1% in an FSES can enhance EMPO by 1.536%. The high coefficient value suggests the huge potential benefits of good family conditions for disabled individuals.

### 4.3. Moderating effect analysis: role of family socioeconomic status in deprivation-employment relations

Our second main interest lies with the moderating role of FSES on IDMD-EMPO relations, which we will turn to now. As shown in Table 5, there is a significant moderating effect of FSES on IDMD-EMPO relations (interaction term = 3.142, *p* < 0.001). Consequently, *H2* is supported. To be explicit, the positive interaction term denotes that FSES positively moderates the relationship between IDMD and EMPO. That is, despite the potential damage of deprivation in enhancing employment for disabled people, family conditions could mitigate such damage by a large amount.

**Table 5:** Results of the moderating effect.

	(1)	(2)	(3)
IDMD	−4.199***		−1.636**
	(0.246)		(0.364)
FSES		2.377***	1.536***
		(0.136)	(0.364)
IDMD*FSES	8.749***	0.014	3.142**
	(0.711)	(0.485)	(1.346)
Control variables	Yes	Yes	Yes
Constant	−2.023***	−3.164***	−2.729***
	(0.080)	(0.343)	(0.0.185)
*F* test	285.97	288.99	254.19
	[0.000]	[0.000]	[0.000]
*R* square	0.452	0.269	0.457

### 4.4. Heterogenous effect analysis: heterogenous characteristics between different disabled population groups

Table 6 further reveals that the impact of FSES is heterogeneous among different disabled population groups. First, FSES has the strongest impact on EMPO from group 4 (high IDMD − low FSES). The coefficient is 14.470 at the value of *p* < 0.01, which means that a 1% increase in FSES leads to a 14.470% increase in the EMPO.

**Table 6:** Results of the moderating effect analysis based on different disabled population groups.

Variables	Groups							
	Low IDMD-high FSES		Low IDMD-low FSES		High IDMD-high FSES		High IDMD-low FSES	
	(1)	(2)	(3)	(4)	(5)	(6)	(7)	(8)
IDMD	−0.0609	−0.157	−0.0597	−1.007	−0.278*	−0.693	−0.553**	−3.853***
	(0.0782)	(0.365)	(0.127)	(0.693)	(0.145)	(0.704)	(0.259)	(1.021)
FSES	1.794***	2.003**	2.594***	6.144**	1.737***	2.506*	3.016***	14.47***
	(0.110)	(0.781)	(0.333)	(2.577)	(0.117)	(1.280)	(0.291)	(3.440)
IDMD*FSES		0.159		2.791		0.722		10.79***
		(0.589)		(2.009)		(1.198)		(3.229)
GEN	0.0115	0.0117	0.0368	0.0318	−0.000650	−0.00156	0.00767	−0.00874
	(0.0450)	(0.0450)	(0.0981)	(0.0981)	(0.0512)	(0.0512)	(0.0895)	(0.0889)
PLAB	−0.0288	−0.0282	−0.149**	−0.146**	0.0186	0.0185	−0.184***	−0.187***
	(0.0332)	(0.0333)	(0.0696)	(0.0696)	(0.0367)	(0.0367)	(0.0633)	(0.0628)
HK	0.640*	0.641*	0.0391	0.0313	0.936**	0.928**	0.252***	0.265***
	(0.378)	(0.378)	(0.144)	(0.144)	(0.421)	(0.422)	(0.0941)	(0.0935)
POP	−0.00364	−0.00346	0.00662	0.00427	−0.0239*	−0.0231*	0.0407*	0.0401*
	(0.0140)	(0.0141)	(0.0260)	(0.0260)	(0.0136)	(0.0137)	(0.0243)	(0.0241)
PDIS	−0.00702	−0.00657	0.0249	0.0252	0.0171	0.0171	0.0351*	0.0371**
	(0.0111)	(0.0113)	(0.0198)	(0.0198)	(0.0106)	(0.0106)	(0.0182)	(0.0181)
Constant	−2.765***	−2.890***	−3.278***	−4.469***	−2.741***	−3.188***	−4.173***	−7.681***
	(0.138)	(0.482)	(0.232)	(0.888)	(0.187)	(0.765)	(0.313)	(1.095)
Observations	687	687	528	528	554	554	648	648
R-squared	0.309	0.309	0.124	0.127	0.330	0.331	0.202	0.216

Second, FSES significantly promotes EMPO for disabled people from group 4 (coefficient = 10.79, *p* < 0.01) but not for disabled people from other groups. For group 4 disabled people, a 1% improvement in FSES could lead to a 10.79% increase in their EMPO. A possible explanation for the above findings is that people with disabilities from families with higher socioeconomic levels are perceived as independent and responsible for their destinies. In contrast, people with disabilities from families with lower socioeconomic levels are dependent on others (71–73).

Third, it is worth noticing that the coefficient of the FSES and the interaction term in the group 4 population are both larger than the coefficient of IDMD. Such finding reveals that enhancing employment through family-level support is more effective than individual-level support for people with disabilities.

## 5. Discussion

### 5.1. Deprivation-employment relations: the poverty trap?

**Figure 4:**
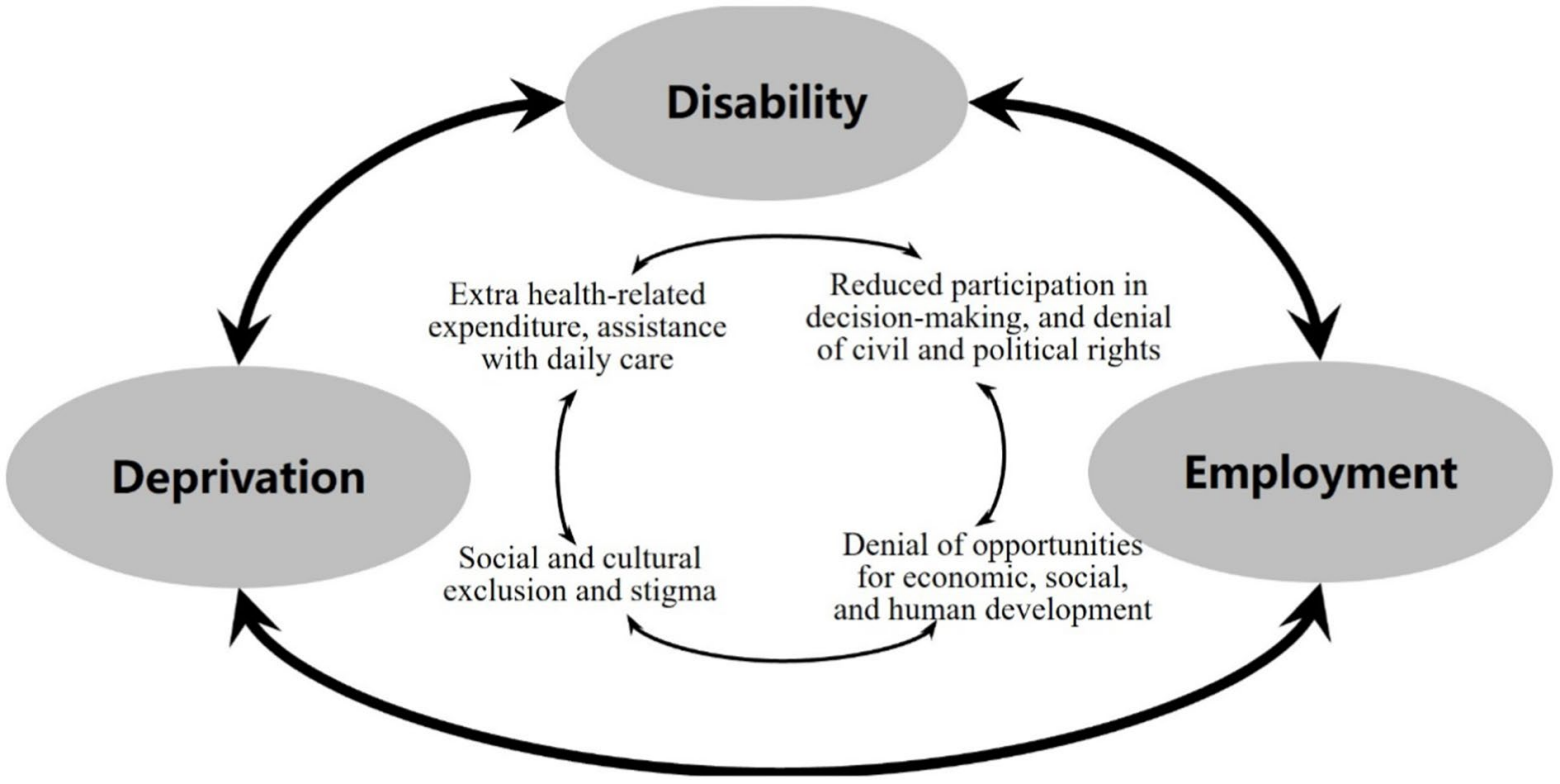
The poverty trap caused by deprivation-employment relations.

This study highlights the significant impact of deprivation on the employment of persons with disabilities, complementing previous research that emphasized the crucial role of employment as a determinant of deprivation. The findings of this study underscore a noteworthy fact: disabled individuals face a poverty trap resulting from the interplay between deprivation and employment (Figure 4). On the one hand, the study reveals that unemployment among disabled individuals signifies the denial of opportunities for economic, social, and human development (74). Consequently, persons with disabilities experience heightened levels of poverty and deprivation while incurring additional costs associated with their disabilities. These costs include health-related expenses, daily care assistance, and transportation, all of which further contribute to their poverty (54). On the other hand, when disabled individuals face severe deprivation, such as social and cultural exclusion and stigma (75), they are at an increased risk of unemployment due to limited access to the labor market. This, in turn, reduces their participation in decision-making processes and denies them civil and political rights (76). These interconnected dynamics create a vicious cycle of poverty and perpetuate marginalization among persons with disabilities.

The planning and development of persons with disabilities, specifically in meeting their fundamental requirements, is intrinsically connected to the imperative of emphasizing their employment. Consequently, there exists a critical necessity to dismantle barriers to employment and economic security faced by individuals with disabilities. Enhancing the labor market participation of people with disabilities yields benefits not only for the individuals but also for their families, society at large, and national development. This is because the development of human resources among disabled individuals is equally vital as the development of human resources among the non-disabled population. Therefore, active participation in discussions aimed at improving the welfare of disabled individuals and society as a whole is imperative.

### 5.2. Dismantling the poverty trap for all: integrating family socioeconomic status into deprivation-employment relations

Previous research has predominantly emphasized individual heterogeneity among disabled individuals, often overlooking the influence of the family environment in which they reside (77). The former pertains to the deprivation of social participation experienced by disabled individuals due to their illnesses and health conditions. Conversely, the latter addresses the significance of incorporating planning and policy-making strategies to enhance the economic and social advantages available to disabled individuals, considering the diverse family contexts in which they live.

**Figure 5:**
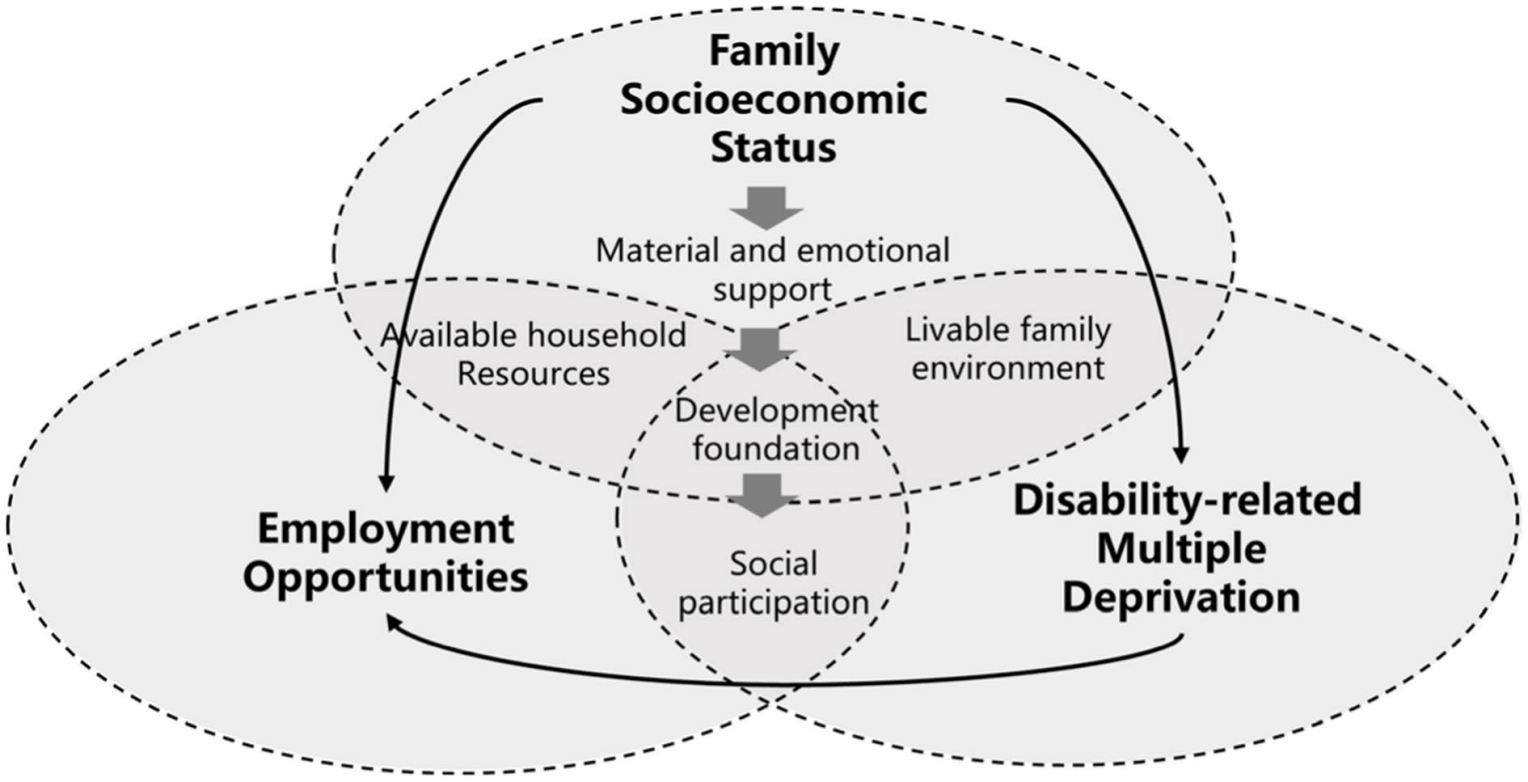
Integrating family socioeconomic status into deprivation-employment relations.

The family socioeconomic status emerges as a crucial determinant of employment outcomes for disabled individuals, considering it is one of the most significant environments in which they reside. Our findings indicate that disabled individuals from families with higher socioeconomic status tend to have better access to resources, including educational opportunities, a supportive marital environment, and social capital (17). These findings align with previous research that highlighted the positive impact of pre-college urban hukou status and a proxy for the father’s education on educational and employment outcomes for graduates (78). Consequently, higher socioeconomic class within the family context translates into increased employment opportunities for their children. Furthermore, it is essential to adopt an intergenerational perspective, as the poverty experienced during youth is often interconnected with parental poverty and childhood deprivation, which can persist throughout the lives of young individuals and their households (79). For disabled individuals, the absence of a livable family environment, such as inadequate healthcare services and inaccessible transportation conditions, exacerbates their multiple deprivations, encompassing education, health, marriage, and other domains (54). Families in better socioeconomic situations may actively seek information about available benefits for persons with disabilities, assist in their application process, and aid in managing their financial resources such as wages or benefits (Figure 5).

The results further uncover how family socioeconomic status could moderate the deprivation-employment relations, especially for the most vulnerable groups of disabled individuals (high IDMD − low FSES). The findings indicate that these highly vulnerable groups exhibit greater sensitivity to both the material and emotional support provided by their families, which serves as the foundation for their personal development and enhances their social participation. These findings underscore the inadequacy of solely focusing on individual-level support for individuals with disabilities without considering their familial circumstances. As a result, this study suggests that disabled individuals from families with more favorable socioeconomic status could benefit from individual-level support. In contrast, disabled individuals from families with lower socioeconomic status are more likely to escape the cycle of poverty through family-level support.

## 6. Conclusion and policy implications

In general, the findings support the research framework and hypothesis. Firstly, the study adds to previous research by revealing that disability-related deprivation restricts employment opportunities for disabled individuals, thus complementing existing studies that highlight employment as a crucial factor contributing to deprivation (80). Building upon these findings, the study further emphasizes the significant issue of disabled individuals being caught in a poverty trap resulting from the interplay between deprivation and employment. Secondly, the study underscores that socioeconomic support from families can effectively alleviate disability-related deprivation and subsequently mitigate the negative impact of deprivation on employment among people with disabilities. This finding emphasizes the importance of family-level support, expanding on previous studies exploring various dimensions of support, such as healthcare, financial assistance, daily care, and emotional support (54). Thirdly, the moderating role of family socioeconomic status is most prominent among the high IDMD and low FSES disabled population groups, indicating that the most vulnerable groups are particularly sensitive to family support.

The empirical evidence from this study has several implications for planning and governance. Firstly, there is a critical need to break down the poverty trap caused by deprivation-employment relations. Providing more employment opportunities and improving the labor market environment for people with disabilities is helpful for disabled people to break the cycle of poverty. Secondly, disability-related policies and funding should not only focus on the individual level but also their families, such as flexible employment opportunities for family members and provision of education resources. It helps to reduce the economic and care burden on families due to disability, break the intergenerational transmission of poverty and alleviate job deprivation for disabled people. Thirdly, this study urges against a one-size-fits-all policy for disabled people with different individual and family conditions. It is more possible for vulnerable groups of individuals with high IDMD but low FSES to escape the poverty trap through family-level rather than individual-level support.

The study has reported some significant results, but it is not without limitations. Firstly, although the neighborhood is already a highly detailed unit for spatial research, the family socioeconomic status of disabled people is still relatively rough to measure. Secondly, the measurement of employment for disabled people used intensive employment opportunities in this study. However, other modes of employment, such as self-employment and enterprise recruitment, should also be included to measure the employment of disabled people accurately. Follow-up studies, therefore, should conduct disability-related surveys and interviews, and pay more attention to their family environment. Furthermore, although IDMD, FSES and EMP are measured in spatial units, the regression analysis is not spatial-based. Future studies would benefit from using a spatial measurement model to reveal the heterogeneous spatial effect.

## Data availability statement

The datasets presented in this article are not readily available because the relevant data require confidentiality agreements. Requests to access the datasets should be directed to Tianjin Municipal Bureau of Statistics.

## Author contributions

NQ: conceptualization and writing – original draft. YJ: data curation and visualization. ZS: methodology and formal analysis. MD: writing – original draft and supervision. All authors contributed to the article and approved the submitted version.

## Funding

This work was supported by Natural Science Foundation of Shandong Province (grant number: ZR2023QE242).

## Conflict of interest

The authors declare that the research was conducted in the absence of any commercial or financial relationships that could be construed as a potential conflict of interest.

## Publisher’s note

All claims expressed in this article are solely those of the authors and do not necessarily represent those of their affiliated organizations, or those of the publisher, the editors and the reviewers. Any product that may be evaluated in this article, or claim that may be made by its manufacturer, is not guaranteed or endorsed by the publisher.

## Footnotes

^1^The Disabled Persons’ Federation is a public institution that participates in the administration of civil servants and is an organization that represents and addresses disabled people’s issues in China.

^2^Fang.com, founded in 1999, is a professional online platform for the real estate home industry covering the areas of new, used, rental, home, and real estate research, with current data for 24 countries and 658 cities worldwide.
